# Vitamin B12—Multifaceted In Vivo Functions and In Vitro Applications

**DOI:** 10.3390/nu15122734

**Published:** 2023-06-13

**Authors:** Krzysztof Halczuk, Julia Kaźmierczak-Barańska, Bolesław T. Karwowski, Aleksandra Karmańska, Marcin Cieślak

**Affiliations:** Food Science Department, Faculty of Pharmacy, Medical University of Lodz, ul. Muszynskiego 1, 90-151 Lodz, Poland; krzysztof.halczuk@umed.lodz.pl (K.H.); julia.kazmierczak-baranska@umed.lodz.pl (J.K.-B.); boleslaw.karwowski@umed.lodz.pl (B.T.K.); aleksandra.karmanska@umed.lodz.pl (A.K.)

**Keywords:** vitamin B12, genome stability, ROS, DNA methylation

## Abstract

Vitamin B12 plays a key role in DNA stability. Research indicates that vitamin B12 deficiency leads to indirect DNA damage, and vitamin B12 supplementation may reverse this effect. Vitamin B12 acts as a cofactor for enzymes such as methionine synthase and methylmalonyl-CoA mutase, which are involved in DNA methylation and nucleotide synthesis. These processes are essential for DNA replication and transcription, and any impairment can result in genetic instability. In addition, vitamin B12 has antioxidant properties that help protect DNA from damage caused by reactive oxygen species. This protection is achieved by scavenging free radicals and reducing oxidative stress. In addition to their protective functions, cobalamins can also generate DNA-damaging radicals in vitro that can be useful in scientific research. Research is also being conducted on the use of vitamin B12 in medicine as vectors for xenobiotics. In summary, vitamin B12 is an essential micronutrient that plays a vital role in DNA stability. It acts as a cofactor for enzymes involved in the synthesis of nucleotides, has antioxidant properties and has potential value as a generator of DNA-damaging radicals and drug transporters.

## 1. Chemical Structure and Biosynthesis of Cobalamin

**Figure 1:**
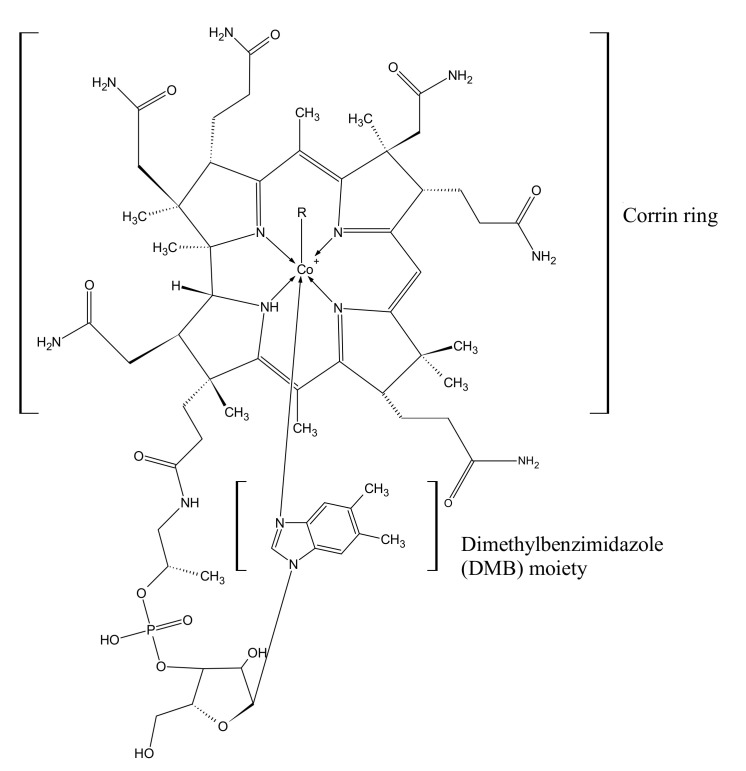
Chemical structure of cobalamins. Depending on the ligand (R), there are 6 chemical compounds of the cobalamin family: CN—cyanocobalamin (CNCbl); CH_3_—methylcobalamin (MeCbl); 5-deoxyadenosine—adenosylcobalamin (AdoCbl); H_2_O—aquacobalamin (AqCbl); OH—hydroxocobalamin (-OHCbl); glutathion—glutathionylcobalamin [1,2].

Cobalamin (Cbl) has been called the most beautiful cofactor in nature, and it is certainly the most chemically complex cofactor of natural origin. The main element of the cobalamin structure is the corrin ring with a central cobalt ion (Figure 1). Depending on the degree of oxidation of cobalt, it can form four to six bonds, four of which are always bound to the nitrogen atoms of the corrin ring. The fifth bond binds to dimethylbenzimidazole (DMB) moieties, and the sixth bond to various ligands, from which cobalamin takes its specific name [1].

The biologically active forms in the human body are methylcobalamin (MeCbl) and adenosylcobalamin (AdoCbl). In addition, cyanocobalamin (CNCbl), as the most durable chemical form, is used in the pharmaceutical industry as an active substance for the production of drugs and dietary supplements [2].

The chemical structure of CNCbl and AdoCbl, i.e., vitamin B12, was first described by Dorothy Hodking in 1955 and 1961, based on X-ray crystallography, for which she was awarded the Nobel Prize in chemistry [3]. The structure of cobalamins is shown in Figure 1.

Cobalamin biosynthesis is limited to some prokaryotic organisms. Approximately 30 enzymatic reactions are involved in the synthesis of vitamin B12. Eukaryotes do not synthesize cobalamin de novo, and its metabolism is limited to methionine synthase (MS) and methylmalonyl-CoA mutase (MUTmCoA) [4].

The corrin ring, the main part of cobamides, can be synthesized de novo by some bacteria and archaea through the aerobic or anaerobic pathway. These processes are already well described [5]. At the same time, many bacteria are able to recover vitamin B12 from cobamides by replacing the fifth ligand (which is usually the DMB moiety) base binding to cobalt. Unfortunately, mammals, including humans, must obtain cobalamin from external sources [4].

## 2. Sources and Absorption of Vitamin B12

**Figure 2:**
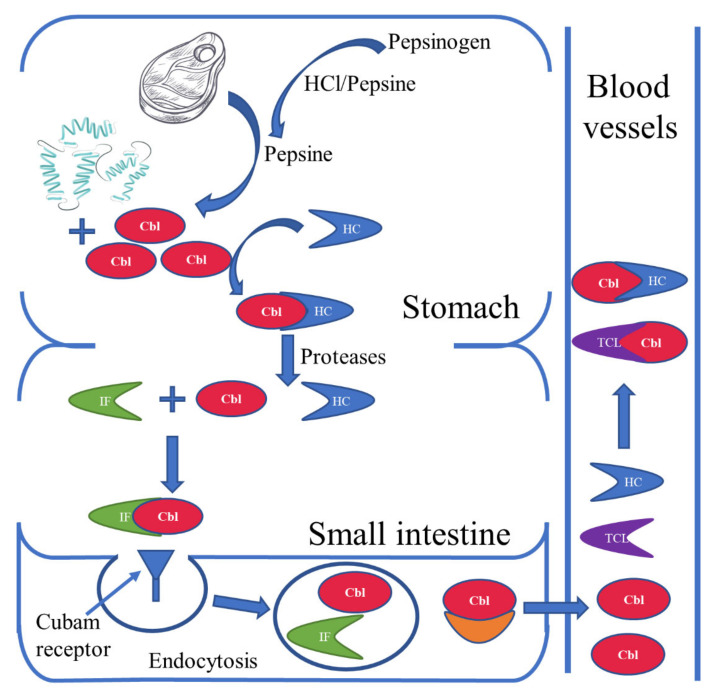
Absorption of vitamin B12. Pepsinogen is converted to pepsin by hydrochloric acid or by pepsin (autoactivation). Pepsin releases cobalamins (Cbl) bound to proteins from food. The released cobalamins bind in an acidic environment with haptocorin (HC), which are transported in this form to the small intestine. At the same time, intrinsic factor (IF) produced by the parietal cells of the stomach enters the intestine. In the duodenum, the Cbl-HC complex is broken down and a new Cbl-IF complex is formed. The cobalamins in this complex are recognized by cubam receptors located in the distal ileum and absorbed by endocytosis. In intestinal villus cells, the lysosomes break down the Cbl-IF complex and release Cbl via, inter alia, basolateral multidrug-resistance protein 1 (MRP1) into the bloodstream. In the blood, Cbl is bound to one of two proteins: HC or transcobalamin (TCL). Importantly, the main source of active Cbl for cells is the Cbl-TCL complex, which is much more easily taken up by cells than Cbl-HC. It should be remembered that, apart from the described mechanism of absorption, active Cbl is also absorbed by passive diffusion along the entire length of the small intestine [1,7,8,9].

Among humans, the primary source of vitamin B12 is considered to be food of animal origin. Some bacteria that make up the human intestinal flora have the ability to synthesize vitamin B12, but its bioavailability in this case is limited. The availability of vitamin B12 produced by intestinal bacteria depends, among other things, on the location of the bacteria in the appropriate section of the intestine, with vitamin B12 being absorbed in the small intestine; the ratio of the amount of cobalamin-producing bacteria to the amount of cobalamin-consuming bacteria; and the presence of diseases caused by bacteria [1,6]. The absorption of vitamin B12 is shown in Figure 2.

Foods high in vitamin B12 include liver, beef, lamb, eggs, milk and dairy products [10]. Its absorption and availability varies depending on the amount and quality of protein consumed. Culinary preparation, especially the heat treatment of meat and milk, can reduce vitamin B12 content by up to 45% [9]. Liver and kidney contain >10 µg/100 g, but when a meal contains more than 2 µg of vitamin B12, absorption decreases [11]. Milk and dairy products have lower vitamin B12 levels than meat. One serving of milk, cheese (hard variety, 60 g) or yogurt (150 g) provides 1.6–4.3 µg/d of vitamin B12 [12].

With normal absorption, the body binds about 50% of a single 1 µg dose, but only 20% of a 5 μg dose and 5% of a 25 μg dose [13]. This is related to the limited number of IF receptors on the enterocyte membrane; these receptors regenerate over a period of about four to six hours, during which time, the absorption of vitamin B12 is limited [14]. To maximize vitamin B12 absorption, its intake should be spread throughout the day [15]. The relationship between the bioavailability of vitamin B12 and the dose was confirmed by Doets et al. in their 2013 systematic review and confirmed by research of by Devi et al. in 2020. The lower the administered dose, the higher the bioavailability of vitamin B12 [16,17]. At the same time, 1–5% of the oral dose of vitamin B12 is passively absorbed along the entire length of the digestive tract, which justifies the treatment of vitamin B12 deficiency with oral preparations [9]. 

Allen et al. [14] report that 4.5% of vitamin B12 from liver is absorbed (vitamin B12 content 38 µg/100 g) compared to 83% from mutton (3 µg/100 g), 24 to 36% from egg products (0.3–0.94 µg/100 g), 65% from chicken (0.4–0.6 µg/100 g), 55–65% from milk (0.3–0.4 µg/100 g) and 30–42% from fish (3.0–8.9 µg/100 g).

Thus, the bioavailability of vitamin B12 from food is usually determined by its content. An exceptional case is chicken eggs, which demonstrate the low bioavailability of vitamin B12 despite its low content. This is caused by ovalbumin, which inhibits the absorption of vitamin B12. For this reason, chicken eggs are poor sources of vitamin B12 compared to other animal foods [18].

Vitamin B12 levels in adults depend on dietary supply. In healthy individuals, a daily vitamin B12 intake of 1–4 μg is considered sufficient to meet nutritional needs [9]. As the total storage of vitamin B12 in the body is typically 3–5 mg, the effects of chronic vitamin deficiency can appear several years after the last dose [19]. Between 1.4 and 5.1 µg of vitamin B12 is excreted daily [16]. The average daily loss is estimated to be 0.13% of the body’s vitamin B12 content, but this value varies greatly between populations. The amount of excreted vitamin B12 is important for the estimation of the daily requirement for this vitamin [16]. We also presume that the large stores of vitamin B12 in the body combined with such a low daily loss are related to the fact that clinical symptoms of vitamin B12 deficiency do not appear until several years after the cessation of vitamin B12 consumption.

It is worth noting that although vitamin B12 belongs to the group of water-soluble vitamins, it is excreted in both urine and feces (together with bile) [16,20]. 

Vitamin B12 deficiency can also be caused by diseases of the gastrointestinal tract: gastritis, intestinal malabsorption, Crohn’s disease, *Helicobacter pylori* infection, parasite infection and chronic pancreatitis. Other causes include old age, difficulty swallowing and the long-term use of proton pump inhibitor drugs and metformin [14,21,22].

Vitamin B12 deficiency, due to insufficient intake, is seen in low-income, malnourished populations and among vegans, vegetarians and the elderly [23,24]. Pregnant women have an increased metabolic need for vitamin B12, and those on vegetarian or vegan diets should therefore consume fortified foods or use supplements [10,24].

The daily requirement for vitamin B12 ranges from 2 to 4 µg/day, depending on the organization that issues the guidelines (Table 1). The recommended amounts are calculated assuming that healthy people absorb approx. 40 or 50% of the vitamin B12 consumed [16].

Several biomarkers are used to assess the blood levels of vitamin B12. The most common is total vitamin B12 measurement, which measures vitamin B12 bound to both the HC and TC transport proteins; however, this test may mask a true deficiency or incorrectly suggest a deficiency. Additionally, Herbert’s model proposes the use of holotranscobalamin, the active form of vitamin B12, as a marker: a low holotranscobalamin level is believed to be a more reliable indicator of vitamin B12 impairment than a low serum vitamin B12 level. However, some studies indicate that this approach does not take into account vitamin B12 portal circulation. It has also been suggested that in general, the use of a single indicator is insufficient to confirm vitamin B12 deficiency [27,28]. The current International Standards for calibration quantitative tests approved by the WHO are 480 pg/mL for serum vitamin B12 and 107 pmol/l for serum holotranscobalamin [29,30].

Normal ranges and standards for cobalamin and holotranscobalamin in the serum are still being analyzed by scientists. Currently, the most commonly accepted cut-off value for serum cobalamin is <147 pmol/L or <200 pmol/L. Below these values, there is a cobalamin deficiency [31,32]. Normal ranges for holotranscobalamin in healthy people are 35–171 pmol/L. However, the lower and upper limits in plasma may vary between 19 and 42 pmol/l and 134 and 157 pmol/l, respectively, depending on the study [31,32]. Normal reference ranges for cobalamin and holotranscobalamin are not dependent on the age, sex or physiological condition of the patient. However, it has been observed that some physiological conditions or diseases may affect the levels of vitamin B12. For example, during pregnancy, the concentration of serum cobalamin can drop by up to 50%, while holotranscobalamin remains unchanged [33]. Increased levels of cobalamin and transcobalamin occur, for example, in the case of renal failure or alcoholism. Chronic myeloid leukemia may cause a significant increase in the concentration of cobalamin in the blood [31,32,34].

Low vitamin B12 levels can be confirmed by using homocysteine and methylmalonic acid (MMA) as markers [32]. Vitamin B12 deficiency results in an increase in methylmalonic acid levels, due to vitamin B12 being needed as a cofactor in converting MMA to succinyl-CoA, and in elevated homocysteine levels; however, high homocysteine levels are also associated with vitamin B6 deficiency, folic acid deficiency or kidney failure [32,35].

The standard treatment for vitamin B12 deficiency includes intramuscular injections, typically in the form of cyanocobalamin. Since 1–5% of oral vitamin B12 is absorbed via passive diffusion along the entire gastrointestinal tract, vitamin B12 deficiencies could be supplemented via high-dose oral supplementation. CNCbl, OHCbl, AdoCbl and MeCbl can be used for dietary supplements, and CNCbl and OHCbl for food fortification. While cyanocobalamin is the best-studied and most stable form and delivers the highest dose, concerns about the possibility of cyanide accumulation in tissues have reduced its popularity [9]. Anders, Zulfikar and Vogel recommend taking CNCbl orally in the dose of 1000 µg per day for a month, and then 125 to 1000 µg until normal levels are achieved. This is especially recommended for patients in whom injections are contraindicated while on antiplatelet drugs or anticoagulants [36]. Vitamin B12 is also absorbed passively in the nasal mucosa. The bioavailability of vitamin B12 in intranasal application varies and depends on the form used, with typical values being 2–5% for hydroxycobalamin, 2–6% for cyanocobalamin and 20% for methylcobalamin [9].

## 3. Cobalamin and Genome Stability

Methylcobalamin is a cofactor of methionine synthase (MS), which catalyzes the formation of methionine from homocysteine. Methionine is then converted to S-adenosyl-L-methionine (SAM), a universal methyl donor used by methyltransferases (MTs) for the methylation of biomolecules, including DNA [37]. In humans, the methylation of DNA is characterized by the addition of a methyl group at the C5 position of the cytosine. Correct DNA methylation regulates gene expression, is responsible for the imprinting of gametes, blocks the expression of the second copy of the X chromosome in women and inactivates retrotransposons. The improper functioning of methylcobalamin-dependent MS may lead to diseases associated with the following pathophysiological mechanisms: the direct toxicity of metabolites (mainly homocysteine), a deficiency of synthesis products (methionine, SAM and 5-methyltetrahydrofolate), methylation disorders and increased oxidative stress [38]. The main symptoms of homocysteine remethylation disorders include, for example: encephalopathies, cardiomyopathies, anemia, poor weight and height gain (in children), pulmonary hypertension, hemolytic uremic syndrome or repeated thromboembolic events [38]. Much attention has also been paid to the influence of the DNA methylation process in common diseases such as rheumatoid arthritis [39], type 1 diabetes [40] or obesity [41]. At the same time, the effect of vitamin B12 supply on the occurrence of obesity (with a negative correlation between the intake of vitamin B12 and obesity) [42] and the occurrence of metabolic syndrome (similar relationship as above) was confirmed [42]. However, the concentration of vitamin B12 in the serum of patients with rheumatoid arthritis seems to be higher than in healthy people [43].

Studies in humans show that inadequate vitamin B12 intake can lead to the hypomethylation of the genome [44]. Mandaviya et al. identified a series of epigenetic loci dependent on vitamin B12 supply and showed that some regions are negatively correlated with vitamin B12 intake [45]. Taking into account the above studies, it can be assumed that both too low and too high an intake of vitamin B12 can lead to DNA hypomethylation. Changes in the methylation profile of genes can also lead to the development of neoplastic diseases, such as thyroid cancer. The thyroid is a special organ because it is also extremely vulnerable to DNA damage due to its intensive metabolism requiring reactive oxygen species [46,47].

In addition to its function as an enzyme cofactor (MS in cytosolic metabolism and MUTmCoA in mitochondria), vitamin B12 is also a scavenger of reactive oxygen species (ROS), with an effect comparable to superoxide dismutase 1 [48]. The reduced form of vitamin B12 (cob(II)amin) directly captures superoxide anions. In addition, cobalamin hampers the activation of the apoptosis factor (caspase-3) caused by oxidative stress and inhibits cell death [49,50] The right level of vitamin B12 also affects the preservation of the right amount of glutathione, which is a direct antioxidant. This is related to the occurrence of hyperhomocysteinemia, which can be caused by vitamin B12 deficiency, and which causes the depletion of glutathione resources [51,52].

In vitro research is being conducted on the pro-oxidative effects of Cbl and its applications to study the structure of nucleic acids and their interactions with other biomacromolecules (e.g., proteins). It has been found that the photolysis of vitamin B12 can generate DNA-damaging radicals. For example, methylcobalamin illuminated with green light causes the cleavage of the Co-C bond, generating the CH_3_ radical, which effectively breaks the DNA strand [53].

**Figure 3:**
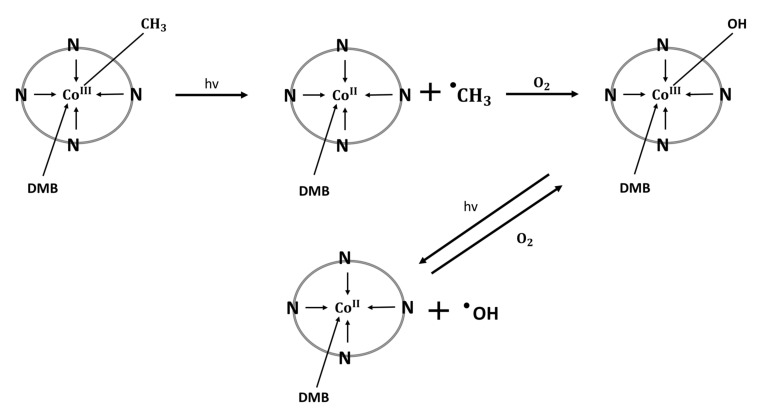
Mechanism of generating methyl and hydroxyl free radicals via photolysis of methylcobalamin and hydroxocobalamin [54]. N: nitrogen atoms belonging to the backbone of the corrin ring; DMB: dimethylbenzimidazole; covalent bond between DMB and corrin ring is not indicated.

Alternatively, in aerobic conditions, MeCbl can be transformed into hydroxycobalamin (Figure 3). The subsequent photolysis of OHCbl leads to the generation of hydroxyl radicals responsible for DNA strand breaks. This phenomenon is minimized under anaerobic conditions or with the use of hydroxyl radical scavengers. These properties are used as an alternative to the Fenton and Haber Weiss reactions in in vitro studies [54]. However, the pro-oxidative properties of vitamin B12 are probably of little physiological importance, as the great majority of in vitro and in vivo studies indicate its antioxidant activity [53,54].

Research is also underway on the use of the photolytic breaking of the Co-C bond in modified cobalamins to transport chemotherapeutic drugs to target cells in the body [55]. For example, the target can be cancer cells that are characterized by rapid proliferation and thus an increased need for nutrients, including vitamin B12. There is a higher concentration of cobalamin in cancer cells compared to normal cells [56]. Marvin et al. proposed an approach for delivering vitamin B12-drug conjugates (in this case, a taxane) to selected tissues. Subsequently, using the properties of the photosensitive Co-C bond, the drug was released in a precisely defined tissue, and upon irradiation with a light of a specific length, adapted to the designed conjugate (650 nm in the Marvin study). This technology requires light to irradiate the tissue and tissue blood supply for the delivery of modified erythrocytes [55].

The mitochondrial form of vitamin B12, AdoCbl, is a cofactor of MUTmCoA, whose main purpose is to provide succinyl-CoA. Succinyl-CoA is a substrate of the Krebs cycle and a succinylation factor for the post-translational modification of proteins (mainly the succinylation of lysine residues) [57]. In the 1960s, it was discovered that the photolysis of AdoCbl under anaerobic conditions leads to the formation of 8, 5’-cycloadenosine [58,59]. This is important from the point of view of DNA damage research, because 8,5′-cycloadenosine is also formed in genomic DNA as a result of, inter alia, oxidative stress or ionizing radiation and can be the cause of various diseases, including neurological disease Xeroderma pigmentosum [60,61].

Vitamin B12 has a proven antioxidant effect and significantly affects the stability of the genome. Decreased levels of MeCbl and AdoCbl impair the redox balance in the body and increase the concentration of TNF-α, resulting in the intensification of DNA damage and inhibition of DNA methylation [62]. However, despite the undoubted protective effect on the genome, cobalamins could potentially destroy DNA both directly via the photolytic generation of DNA-damaging radicals and indirectly via the use of modified cobalamins as vectors for chemotherapeutic drugs in the treatment of cancer.

**Figure 4:**
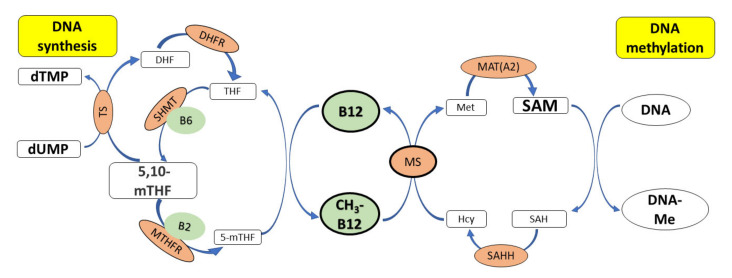
The role of vitamin B12 in synthesis and methylation of DNA. CH3-B12 serves as a cofactor for the methionine synthase (MS), which catalyzes the conversion of homocysteine (Hcy) to methionine (Met). Methionine is further converted to SAM (a donor of –CH_3_ groups during methylation of DNA) by methionine adenosyltransferase. 5,10-mTHF donates a methyl group during the synthesis of dTMP from dUMP, which is catalyzed by thymidylate synthase (TS). Deficiency of vitamin B12 leads to low levels of methionine and SAM and reduced methylation of DNA. SAM also inhibits MTHFR, thus preserving the cellular pool of 5,10-mTHF required for synthesis of dTMP. Deficiency of vitamin B12 results in low levels of SAM and high activity of methylenetetrahydrofolate reductase (MTHFR), which converts 5,10-mTHF to 5-mTHF, finally leading to an increase in the dUMP pool and uracil incorporation into DNA. Legend: 5,10-mTHF—N5,N10-methylenetetrahydrofolate, SAHH—S-adenosylhomocysteine hydrolase, Cys—cysteinę, DHF—dihydrofolate, DHFR—dihydrofolate reductase, dTMP—deoxythymidine monophosphate, dUMP—deoxyuridine monophosphate, Hcy—homocysteine, MAT(A2)—methionine adenosyltransferase, Met—methionine, MS—methionine synthase, MTHFR—methylenetetrahydrofolate reductase, MT—methyltransferase, SAH—S-adenosylhomocysteine, SAM—S-adenosylmethionine, SHMT—hydroxymethyltransferase, THF—tetrahydrofolate, TS—thymidylate synthase, B2—vitamin B2, B6—vitamin B6.

In addition to influencing DNA methylation via MS and SAM, vitamin B12 also indirectly regulates the level of cellular 5,10-methylene tetrahydrofolate (THF), which acts as a methyl group donor during the synthesis of dTMP (Figure 4). Vitamin B12 deficiency can decrease the amount of 5,10-methylene THF and reduce intracellular dTMP levels; this results in dUMP being erroneously incorporated instead of dTMP during DNA synthesis, leading to single- or double-strand breaks, chromosome breaks or micronucleus formation. Thus, vitamin B12 deficiency can cause functional folate deficiency (this has the effect of a reduced supply of folate), which can result in increased DNA damage and abnormal DNA methylation, processes important for cancer development [63,64]. The risk of stomach cancer has been found to be higher with reduced serum vitamin B12 levels. In addition, the adequate intake of folic acid reduces the risk of squamous cell carcinoma of the head and neck and cancer of the mouth and throat, pancreas and bladder [65,66].

A number of observational and clinical studies indicate that vitamin B12 deficiency may be related to increased genotoxicity and suggest that vitamin B12 may have a protective effect against DNA damage. A cross-sectional study in an Australian population (106 participants aged 18–32) found that in women, the frequency of DNA damage (measured as the number of micronuclei) was inversely correlated with the concentration of the plasma vitamin B12. Interestingly, no relationship was found between the concentration of vitamin B12 and DNA methylation. Vitamin B12 supplementation for three months at 3.5× the Recommended Daily Intake (RDI) followed by three months at 10× the RDI resulted in a 25% reduction in the number of micronuclei in participants with an initial DNA damage index above the 50th percentile. The lowest incidence of DNA damage was observed at vitamin B12 concentrations > 300 pmol/L [67]. An inverse correlation between the frequency of DNA damage (number of micronuclei) and vitamin B12 concentration was also observed in men [68,69], with the highest level of DNA damage being observed in individuals with the concentration below 400 pg/L [70].

In addition, children diagnosed with vitamin B12 deficiency also demonstrated a significantly higher frequency of DNA strand breaks. Eight-day supplementation reduced the amount of DNA damage; however, it was still higher than in a control group of children with normal levels of vitamin B12 [71]. Another pediatric study showed that B12 supplementation reduced oxidative stress in children with vitamin B12 deficiency and improved antioxidant parameters, such as total antioxidant status (TAS), total oxidant status (TOS), total thiol (TT) and native thiol (NT) [72]. The above data indicate that in vivo, vitamin B12 has a protective effect on DNA and exhibits antioxidant properties.

Observations of increased genotoxicity in humans subject to cobalamin deficiency were also corroborated in animal studies. The 10-week dietary depletion of vitamin B12 in rats resulted in a 35% reduction in methylcytosine content (DNA hypomethylation) and 105% increase in uracil contents in genomic DNA isolated from colonic mucosa. This observation may provide a link between B12 deficiency and colorectal cancer [73]. Similar results were published by Fernandez-Roig et al., who investigated the effect of transcobalamin receptor (TCblR) deletion on the levels of vitamin B12 and DNA methylation in the mouse brain. TCblR KO mice showed a 90% reduction in vitamin B12 levels and a consequent 44% reduction in DNA methylation [74].

It was also demonstrated that vitamin B12 (particularly when combined with folate) shows antioxidant activity in vivo. The supplementation of rats with cobalamin and folate protected the colon, lung and liver tissues from oxidative DNA damage (measured as 8-hydroxydeoxyguanosine content or DNA fragmentation) induced by azoxymethane [75] or sodium arsenite [76].

The antioxidant properties of vitamin B12 and its protective effect on DNA have also been demonstrated in in vitro studies. The analysis of peripheral blood lymphocytes incubated with paclitaxel (10 µM), an anti-cancer drug with genotoxic and pro-oxidant effects, showed an approximately six-fold increase in the number of DNA breaks and a two-fold increase in 8-hydroxyguanosine levels compared to the control cells. Interestingly, the preincubation of lymphocytes with vitamin B12 (CNCbl at 2.7 mg/mL) protected against DNA damage induced by paclitaxel [77]. Vitamin B12 demonstrated similar antioxidative and DNA protective effects in lymphocytes incubated with pioglitazone: a drug used in the treatment of type 2 diabetes mellitus, which induces DNA strand breaks and increases the level of 8-hydroxyguanosine in lymphocytes [78].

Studies in HeLa cells and human fibroblasts with mutated methionine synthase (a model of cobalamin deficiency) revealed that the depletion of vitamin B12 leads to the accumulation of 5-mTHF and impairs the biosynthesis of dTMP. Cobalamin-depleted HeLa cells also exhibited an increase in DNA double-strand breaks, as evidenced by the increased immunostaining of phosphorylated histone H2AX. This can be explained by the insufficient dTMP pool and erroneously incorporated uracil during DNA synthesis. Interestingly, increased genome instability was not observed in fibroblasts with MS loss of function [79].

## 4. Cobalamin—Regulator of Inflammation and Oxidative Stress

Cbl is an essential factor for cell proliferation, DNA synthesis (an essential cofactor in methylation processes) and mitochondrial metabolism. It also acts as a coenzyme in the metabolism of various amino acids such as methionine, threonine and valine. A reduced form of vitamin B12 (cob(II)alamin) also exhibits antioxidant properties which are realized through multiple mechanisms. Many in vitro studies confirm a reduction in the amount of superoxide ions in the cytosol and mitochondria in the presence of physiological levels of vitamin B12, indicating the direct scavenging of ROS. Cob(II)alamin shows strong ROS-scavenging properties in the nervous system [80]. The antioxidant properties of cob(II)alamin, especially those related to superoxide radical binding, play a key role in its anti-inflammatory and protective effects against oxidative DNA damage [49,81,82].

Other studies indicate that vitamin B12 may also support antioxidant activities by preserving cellular glutathione, a deficiency of which results in the accumulation of hydrogen peroxide [83,84,85]. Vitamin B12 can also exert its indirect antioxidant properties by participating in the metabolism of homocysteine. Excess homocysteine (Hcy) leads to the formation of various ROSs and has been implicated in the pathophysiology of several clinical conditions, such as Alzheimer’s disease, schizophrenia or cardiovascular disease [86,87,88,89,90]. This process is counteracted by vitamin B12, which enables the conversion of homocysteine to methionine [49]. Vitamin B12 deficiency thus disturbs the homeostasis of the cell by shifting it in a pro-oxidative direction, characterized by excess ROSs and oxidative stress. Furthermore, low levels of vitamin B12 favor an increase in the pro-inflammatory cytokine IL-6. The analysis of vitamin B12 and IL-6 levels in peripheral blood mononuclear cells (PBMCs) showed greater basal IL-6 production in patients who had low vitamin B12 levels [91]. Pro-inflammatory cytokines induce inflammation that increases the levels of ROS and can lead to oxidative stress [92]. Elevated ROSs can activate a number of cellular pathways involved in proliferation, tumorigenesis and cancer progression, for example, mitogen-activated protein kinase (MAPK) pathways [93]. A large excess of ROSs may also exert an antitumor effect by inducing oxidative DNA damage and cytotoxicity, which is used in some therapeutic strategies. For example, an ROS increase upon stimulation with tumor necrosis factor (TNFα) activates apoptosis-signal-regulated kinase 1 (ASK1) and induces apoptosis [93,94,95,96].

Inside a cell, cyanocobalamin is converted into the active cofactors methylcobalamin or adenosylcobalamin, which are quickly depleted in rapidly dividing cancer cells. Methylcobalamin is a cofactor for MS, which catalyzes the remethylation of homocysteine to methionine. In addition, vitamin B12 is also needed for the regeneration of folic acid, i.e., methyltetrahydrofolate to tetrahydrofolate. Folates act as coenzymes in the biosynthesis of nucleotides and amino acid metabolism; thus, a vitamin B12 deficiency limits the pool of methionine but also impairs DNA synthesis. In addition, the accumulation of homocysteine promotes oxidative stress, and the trapped methyl group impairs the cell’s ability to transmethylate, resulting in DNA hypomethylation [97]. However, it is unknown whether vitamin B12 levels can be used as a risk factor for cancer prediction: some meta-analyses indicate that elevated vitamin B12 plasma levels are positively associated with lung, liver or pancreatic cancer, while others do not [98,99,100].

Due to the extremely high rate of proliferation, cancer cells have an increased need for nutrients and tend to accumulate cobalamin, which makes it an interesting carrier for other anticancer drugs (such as cis-Pt) or imaging agents [101,102]. Some studies indicate that vitamin B12 used in combination therapy may increase the effectiveness of anticancer drugs or reduce their toxicity to healthy cells [77,103,104]. For example, vitamin B12 increases the cytotoxicity of 1,25-dihydroxycholecalciferol (vitamin D3) against various types of cancer cells, enhances the antiproliferative effect and induces apoptosis dependent on the activation of caspase 4 and caspase 8 [105].

Vitamin B12 affects a wide range of functions in the cell and the body, which may explain its activity in the prevention and the course of cancer. There is a considerable correlation between low vitamin B12 levels and increased oxidative stress in healthy subjects [72,106,107]. Meanwhile, there are also studies that show a positive association between elevated vitamin B12 levels and cancer risk in the prostate (OR = 1.17 in the highest vs. lowest quartile of B12 concentration; in the meta-analysis OR = 1.10 for 100 pmol/L increase in B12 concentration) and lungs (OR = 1.15 for a doubling in B12 concentration) [108,109]. An increase in cobalamin in serum is one of the diagnostic criteria for promyelocytic leukemia [110], and high plasma vitamin B12 levels are predictive of poor survival for patients with hepatocellular carcinoma (HCC) [111]. Hence, it should be emphasized that high doses of dietary supplements are not recommended for cancer prevention: nutritional needs should be met through healthy food and a proper diet, especially in healthy people, as this has a better impact on cancer protection than dietary supplements [112].

## 5. Conclusions

Vitamin B12, i.e., a group of chemical compounds called cobalamins, is essential for the proper functioning and metabolism of DNA. Indeed, numerous clinical studies report lower systemic vitamin B12 concentrations to be associated with higher levels of DNA damage and subsequent vitamin B12 treatment to decrease this damage [67,68,69,70,71,72,77,78].

Vitamin B12 not only protects DNA from damage by performing its physiological role as an MS and MUTmCoA cofactor, but it is also a powerful antioxidant itself, reducing oxidative stress, which is the main cause of DNA damage.

Due to the chemical structure and the relatively weak binding of the sixth ligand to cobalt, vitamin B12 can be used as a generator of DNA-damaging radicals. These properties can be used in laboratory preparation, in which the photolysis of cobalamins offers more controlled ROS generation than the Fenton reaction, and in medicine, where cobalamins can be used as vectors for xenobiotics, e.g., in the transport of drugs and their controlled release in cancer tissues.

## Figures and Tables

**Table 1:** Recommended intakes of dietary vitamin B12 around the world. SCF—Scientific Committee on Food; EFSA—European Food Safety Authority; WHO—World Health Organization; FAO—Food and Agriculture Organization [22,25,26].

	EFSA (2015)	WHO/FAO (2004)	SCF (1993)	Poland
Age (years)	>18	≥19	≥19	≥19
Men + Women				
Reference value (μg/day)	4.0	2.4	1.4	2.4
Pregnant Women				
Reference value (μg/day)	4.5	2.6	1.6	2.6

## Data Availability

No new data were created or analyzed in this study. Data sharing is not applicable to this article.
